# Measurement Invariance and Latent Mean Differences in the Reynolds Intellectual Assessment Scales (RIAS): Does the German Version of the RIAS Allow a Valid Assessment of Individuals with a Migration Background?

**DOI:** 10.1371/journal.pone.0166533

**Published:** 2016-11-15

**Authors:** Jasmin T. Gygi, Elodie Fux, Alexander Grob, Priska Hagmann-von Arx

**Affiliations:** Department of Psychology, University of Basel, Basel, Switzerland; IRCCS Istituto Auxologico Italiano, ITALY

## Abstract

This study examined measurement invariance and latent mean differences in the German version of the Reynolds Intellectual Assessment Scales (RIAS) for 316 individuals with a migration background (defined as speaking German as a second language) and 316 sex- and age-matched natives. The RIAS measures general intelligence (single-factor structure) and its two components, verbal and nonverbal intelligence (two-factor structure). Results of a multi-group confirmatory factor analysis showed scalar invariance for the two-factor and partial scalar invariance for the single-factor structure. We conclude that the two-factor structure of the RIAS is comparable across groups. Hence, verbal and nonverbal intelligence but not general intelligence should be considered when comparing RIAS test results of individuals with and without a migration background. Further, latent mean differences especially on the verbal, but also on the nonverbal intelligence index indicate language barriers for individuals with a migration background, as subtests corresponding to verbal intelligence require higher skills in German language. Moreover, cultural, environmental, and social factors that have to be taken into account when assessing individuals with a migration background are discussed.

## Introduction

Migration has increased in recent decades in Europe [1]. For German-speaking countries, for example, the latest numbers reveal that 25% of the Swiss population [2] and 21% of the German population [3] have a migration background. The majority of immigrants originated from other European countries, including, for example, Italy and Turkey, while a smaller percentage originated from non-Western countries. The immigration of non-German-speaking individuals into Switzerland and Germany leads to more people seeking psychological assessment in a foreign-language environment in the respective countries.

Intelligence is one of the constructs most often studied in psychological practice [4]. Yet in individuals with a migration background, language barriers can lead to difficulties during assessment [5–7] such as misunderstanding of instructions or difficulties in articulating a verbal response. Thus, lower test scores in individuals with a migration background are particularly evident in tasks with high language requirements and their test performance increases with decreasing language dependence of the tasks [6–9]. Moreover, cultural and environmental factors may adversely affect test performance of individuals with a migration background [10]. For example, individuals with a migration background may be less familiar with the type of tasks used in performance assessment [11, 12]. In addition, many immigrant groups are confronted with negative achievement stereotypes, which may create increased performance pressure [13]. Furthermore, studies have shown that immigrant children’s parents who have limited language skills in the dominant language are less involved in schooling, which in turn may negatively affect their children’s performance [14, 15]. Consequently, in intelligence tests, individuals with a migration background may not be able to show their full potential, which can result in test scores that underestimate intelligence [5–7, 11, 12]. As high-stakes decisions are made on the basis of intelligence test scores, underestimated intelligence test scores may have negative consequences in, for example, education and employment. Regarding school-related decisions, underestimated intelligence test scores may lead to erroneous placement in special education services [16, 17]. In workplace situations, negative stereotypes may negatively influence performance ratings [18] and underestimated intelligence test scores may lead to not getting hired (e.g., [4]). Understanding how migration status influences test scores is therefore crucial to correctly interpreting the results.

To assess test score differences in individuals with and without a migration background, it is essential to examine measurement invariance prior to any interpretation of group differences [19]. Measurement invariance refers to the assumption of comparable relationships between items and their respective latent variables across groups [20]. When measurement invariance holds, researchers can validly compare statistical results, such as latent means, across these groups [21].

One currently used intelligence test is the Reynolds Intellectual Assessment Scales (RIAS) [22]. The RIAS is an individually administered test for individuals aged 3 to 90-plus years developed in the United States and recently adapted to different language groups including Danish [23], German [24], and Spanish [25]. The RIAS is designed to measure general intelligence and its two components, verbal and nonverbal intelligence. Additionally, the RIAS provides a measure of memory. Performance on the RIAS has been shown to be independent of motor coordination, visual-motor speed, and reading skills. Also, its administration, scoring, and interpretation have been described as user friendly [26–28]. However, we know of no study that has examined the RIAS intelligence factor structure across individuals with and without a migration background and whether these groups of individuals achieve comparable RIAS intelligence test scores.

**Fig 1:**
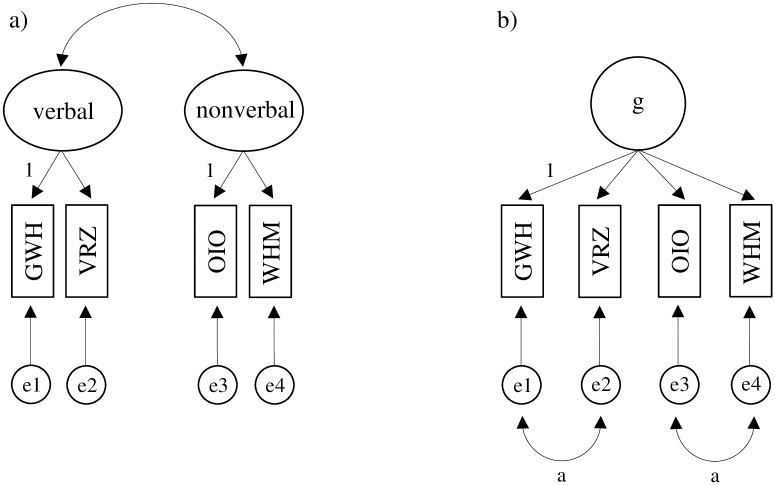
The Two-factor Structure (a) and Single-factor Structure (b) of the RIAS. g = general intelligence; GWH = Guess What; VRZ = Verbal Reasoning; OIO = Odd-Item Out; WHM = What’s Missing; e1-e4 = residuals; a = covariances of the residuals were set to be equal across groups.

The intelligence factor structure of the RIAS is based on Carroll’s [29] three-stratum theory. On Stratum 1 the RIAS comprises two verbal subtests (Guess What, Verbal Reasoning) and two nonverbal subtests (Odd-Item Out, What’s Missing?). On Stratum 2 the subtests are combined to create two factors, the Verbal Intelligence Index (VIX) and the Nonverbal Intelligence Index (NIX), which serve as indicators of crystallized and fluid intelligence, respectively. Stratum 3 comprises a Composite Intelligence Index (CIX), which is obtained by combining the VIX and NIX and which reflects general intelligence, g. A Composite Memory Index (CMX) on Stratum 2, which consists of two supplemental Stratum 1 subtests, is not integrated into the measure of general intelligence. On the basis of these theoretical assumptions, Reynolds and Kamphaus [22] suggested a two-factor intelligence structure with four subtests measuring two factors, verbal and nonverbal, (Fig 1a). Another structure assessed by Reynolds and Kamphaus [22] was a single-factor structure with four subtests measuring general intelligence (Fig 1b).

The RIAS intelligence factor structure proposed by Reynolds and Kamphaus [22] (Fig 1a) has been supported in the English, Danish, German, and Spanish RIAS versions by using confirmatory factor analysis (CFA) and standardization samples [22–25] as well as referred students in the English version [30]. Further, studies using CFA procedures also found support for the single-factor structure in the standardization samples of the four RIAS versions [31] as well as in typically developing and referred samples in the English version [32]. Additionally, studies using exploratory factor analysis have found evidence for a single-factor structure (Fig 1b) for the English RIAS standardization sample [33] and for referred samples [34–36]. While the theoretically proposed RIAS intelligence factor structure was supported in standardization and referred samples, it is unknown whether there is measurement invariance across groups with and without a migration background.

The main goal of the present study was to assess measurement invariance and mean differences in the German version of the RIAS in individuals with and without a migration background. The German version of the RIAS was standardized in Switzerland and Germany. Both countries have a high percentage of individuals with a migration background of over 20% of the population [2, 3]. Therefore, it is essential to ensure measurement invariance of diagnostic instruments such as the RIAS, so that examiners can rely on the validity of test results for individuals with a migration background. In the current study we addressed the following research questions: (a) Are the intelligence factor structures (i.e., single-factor structure and two-factor structure) of the RIAS invariant across individuals with and without a migration background, and (b) do individuals with and without a migration background differ in latent means and variances in the RIAS intelligence factors (the VIX, NIX, and CIX)?

First, we conducted single-group CFAs to determine model fit for the RIAS intelligence factor structures depicted in Fig 1a and 1b for both groups separately. Second, we ran a multi-group CFA for both RIAS intelligence factor structures to assess measurement invariance across groups. We hypothesized that measurement invariance would hold across groups. When invariance held, we examined invariance of the latent variances and latent means across groups. We assumed that individuals with a migration background would have lower means in the latent variables VIX, NIX, and CIX than individuals without a migration background. We further assumed that lower latent means would in particular be evident in the RIAS VIX, drawing on the assumption that language barriers might have led to lower test performance as the verbal subtests require higher skills in German language than the nonverbal subtests [6–9].

## Method

### Participants

The study included 632 individuals with and without a migration background (each *n* = 316) from the standardization sample of the German version of the RIAS (standardization sample *N* = 2,145) [24]. This study was carried out in accordance with the recommendations of the Declaration of Helsinki, and it was approved by the Ethics Committee of Basel. Written informed consent was obtained from all subjects. For the children, parents gave written informed consent and assent was obtained from each child prior to the start of the study. Individuals with and without a migration background were matched in sex and age. Sex was equally distributed, with 48% female and 52% male, according to chi-square tests (*p* > .05). Subjects were between the ages of 3 and 99 years (*M = * 15.79, *SD* = 16.81). The frequencies of individuals in different age ranges are provided in S1 Table. Migration background was defined as speaking German as a first or a second language: Individuals without a migration background all spoke German as their first language, whereas individuals with a migration background all spoke German as a second language. In our study, 88% of individuals with a migration background spoke a language from a Western, European country as their first language, while 12% of the immigrants spoke a language from a non-Western country as their first language. First languages most often named by individuals with a migration background were Turkish (21%), Italian (8%), Serbian (6%), and Spanish (5%). S2 Table gives a detailed overview of all foreign first languages. The distribution of Western and non-Western immigrants represents the immigrant population in Switzerland and Germany well [2, 3], and thus all individuals with a migration background were included in the following analyses. However, there are studies that revealed group differences in intelligence across individuals from Western and immigrants from non-Western countries [37]. In the present study, the sample of immigrants from non-Western countries was not large enough to analyze such group differences. However, we also performed all analyses with immigrants from Western European countries only. As the results remained stable, we present results only for the whole sample including immigrants from both Western and non-Western countries, as we believe this distribution is more representative of the immigrant populations in Switzerland and Germany.

### Measures

The RIAS [22] is an individually administered intelligence test. Its German version [24] was standardized for individuals aged 3 to 99 years in Germany and Switzerland (*N* = 2,145) in 2011 and 2012. The RIAS is composed of four intelligence subtests, which together constitute the CIX. The CIX can be further divided into two indices, represented by two subtests each: The verbal, VIX, comprises the subtests Guess What (GWH), in which individuals are asked to identify an object or a concept through the use of verbally presented clues, and Verbal Reasoning (VRZ), in which individuals are asked to complete verbal analogies. Therefore, the verbal subtests require high language skills. The nonverbal, NIX, comprises the subtests Odd-Item Out (OIO), in which individuals are asked to identify a picture that does not go with others, and What’s Missing? (WHM), in which individuals are required to identify the missing element in a presented picture. The CIX is based on the sum of the *T* scores (*M* = 50; *SD* = 10) of the four intelligence subtests. Reliability for the German RIAS is high for both groups with Cronbach’s α > .90 for the subtests and α > .95 for intelligence indexes.

### Data analysis

To evaluate measurement invariance and invariance of latent variances and means across individuals with and without a migration background we used SPSS AMOS version 22 [38]. Correlation matrices with means and standard deviations of the subtest *T* scores were used as the input data file (see Table 1).

**Table 1:** Correlations Between Reynolds Intellectual Assessment Scales Subtests and Indexes for Individuals With and Without a Migration Background With Means and Standard Deviations.

Subtest or index	GWH^a^	VRZ^a^	OIO^a^	WHM^a^	VIX^b^	NIX^b^	CIX^b^
Without migration background							
GWH	1.00						
VRZ	.58	1.00					
OIO	.30	.35	1.00				
WHM	.32	.28	.38	1.00			
VIX	.88	.90	.36	.33	1.00		
NIX	.37	.38	.83	.83	.42	1.00	
CIX	.75	.77	.70	.68	.86	.83	1.00
Mean	53.11	53.39	52.21	51.60	105.85	103.50	105.34
*SD*	8.48	9.61	8.85	8.75	13.52	12.46	12.43
With migration background							
GWH	1.00						
VRZ	.65	1.00					
OIO	.37	.43	1.00				
WHM	.31	.32	.57	1.00			
VIX	.93	.88	.43	.34	1.00		
NIX	.38	.42	.89	.88	.44	1.00	
CIX	.77	.77	.78	.72	.85	.85	1.00
Mean	44.29	45.58	47.99	47.61	91.86	96.54	93.44
*SD*	11.28	8.96	10.66	10.28	15.39	15.68	14.92
Cohen’s *d*^c^	1.04	0.81	0.48	0.46	1.03	0.56	0.96

*Note*. *N*_Without migration background_ = 316, *N*_With migration background_ = 316. *SD* = standard deviation; GWH = Guess What; VRZ = Verbal Reasoning; OIO = Odd-Item Out; WHM = What’s Missing; VIX = Verbal Intelligence Index; NIX = Nonverbal Intelligence Index; CIX = Composite Intelligence Index.

^a^*T* score normative mean = 50, *SD* = 10.

^b^Intelligence indexes normative mean = 100, *SD* = 15.

^c^Cohen’s *d* is calculated based on the manifest data.

Following the suggestion of Meade, Johnson, and Braddy [39], we first conducted single-group CFAs to test the two-factor and single-factor structures, depicted in Fig 1, for each group. Second, we conducted a multi-group CFA for both factor structures across the two groups. Different levels of measurement invariance were tested, as described by Meredith [20], Milfont and Fischer [40], Steenkamp and Baumgartner [41], and Widaman and Reise [19]. First, we assessed configural invariance (Model 1). The intercepts of GWH and OIO were fixed to zero across both groups for the two-factor structure, and the intercept of GWH was fixed to zero across both groups for the single-factor structure. Second, we tested metric invariance (known as weak invariance; Model 2) by additionally constraining factor loadings across groups. Third, we examined scalar invariance (known as strong invariance; Model 3), by additionally constraining intercepts across groups. Fourth, we assessed residual invariance (known as strict invariance; Model 4) by additionally constraining error variances across groups. Finally, when scalar invariance held, invariance of the latent factors’ variances and means could be analyzed [19] by constraining factor variances (Model 5) and factor means (Model 6) using the scalar invariance model as baseline.

For the single-factor structure, following Beaujean and McGlaughlin [34], we let the residuals of GWH and VRZ as well as the residuals of OIO and WHM to covary and constrained these two covariances to be equal [36]. We stress that this structure leads to a model empirically equivalent [42] to the two-factor structure as well as to a bifactor structure with a single general factor and two specific factors each measured by two indicators with factor loadings fixed to 1 [43, 44].

Following Hu and Bentler [45], we used a Comparative Fit Index (CFI) of ≥ .95, a McDonald’s Noncentrality Index (Mc) of ≥ .90, and a root mean square error of approximation (RMSEA) of ≤ .06 to evaluate good model fit. Because chi-square test statistics tend to be sensitive to large sample sizes [39], we relied on the test of small difference in fit with *p* < .05 [46] and changes in goodness-of-fit indices with ΔCFI ≤ .002 and ΔMc ≤ .005 [39] to compare the nested models. We accepted model invariance if two of the three difference in fit statistics were within cutoff points.

Additionally, we computed Cohen’s *d* [47] to investigate the effect sizes of the manifest and latent mean differences. A value of *d* = 0.2 is considered a small effect, *d* = 0.5 a medium effect, and *d* = 0.8 a large effect.

## Results

### Single-group CFA

The two-factor (Fig 1a) and single-factor (Fig 1b) RIAS intelligence structures were evaluated for both groups using single-group CFA. As can be seen in Table 2, both structures yielded a good model fit for each group (CFI ≥ .993, Mc ≥ .998, RMSEA ≤ .070), indicating the models to be empirically equivalent [42]. Therefore, both structures were further analyzed to assess invariance of the latent variances and means of verbal and nonverbal intelligence (i.e., for this we analyzed the two-factor structure), as well as general intelligence (i.e., for this we analyzed the single-factor structure). The factor loadings, covariances, and correlations for both RIAS structures are provided in S3 Table.

**Table 2:** Fit Indices for Single-group Confirmatory Factor Analyses Evaluating Two Reynolds Intellectual Assessment Scales Intelligence Factor Structures in Individuals With and Without a Migration Background.

Structure	*df0*	χ^2^	CFI	Mc	RMSEA	90% CI
Two factors^a^						
Without migration background	1	2.556	.993	.998	.070	[.000, .183]
With migration background	1	0.438	1.000	1.000	< .001	[.000, .129]
Single factor^a^						
Without migration background	1	2.556	.993	.998	.070	[.000, .183]
With migration background	1	0.438	1.000	1.000	< .001	[.000, .129]

*Note*. *N*_Without migration background_ = 316, *N*_With migration background_ = 316. CFI = Comparative Fit Index; Mc = McDonald’s Noncentrality Index; RMSEA = root mean square error of approximation; CI = confidence interval.

^a^Includes four subtests.

### Multi-group CFA

Measurement invariance across both groups was analyzed using multi-group CFA. For the two-factor structure (Fig 1a) results are shown in Table 3. Configural, metric, and scalar invariance did hold across both groups with regard to ΔCFI, ΔMc, and the *p* value of the test of small difference in fit. Residual invariance yielded a poor fit regarding ΔCFI and ΔMc, though the *p* value of the test of small difference in fit was < .05. Hence, we conclude that the variances of the residuals were noninvariant across groups.

**Table 3:** Fit Indices for Multi-group Confirmatory Factor Analysis Evaluating Measurement Invariance of the Two-factor Structure Including the Four Intelligence Subtests Across Individuals With and Without a Migration Background.

Model	*df*	χ^*2*^	CFI	Mc	RMSEA	90% CI	Δ*df*	Δχ^2^	ΔCFI	ΔMc	Small diff *p*
1 Configural invariance	2	2.994	.998	.999	.028	[.000, .089]	–	–	–	–	–
2 Metric invariance	4	6.010	.997	.998	.028	[.000, .071]	–	–	–	–	–
2 versus 1	–	–	–	–	–	–	2	3.016	.001	< .001	.221
3 Scalar invariance	6	7.309	.998	.999	.019	[.000, .057]	–	–	–	–	–
3 versus 2	–	–	–	–	–	–	2	1.299	< .001	< .001	.522
4 Residual invariance	10	37.877	.954	.978	.067	[.045, .090]	–	–	–	–	–
4 versus 3	–	–	–	–	–	–	4	30.567	.044	.021	.196
5a Factor variances verbal^a^	7	12.273	.991	.996	.035	[.000, .066]	–	–	–	–	–
5a versus 3	–	–	–	–	–	–	1	4.963	.007	.003	.296
5b Factor variances nonverbal^a^	7	24.161	.971	.986	.062	[.036, .090]	–	–	–	–	–
5b versus 3	–	–	–	–	–	–	1	16.852	.027	.013	.217
6a Factor means verbal^a^	7	139.309	.780	.900	.173	[.149, .199]	–	–	–	–	–
6a versus 3	–	–	–	–	–	–	1	132.000	.218	.099	.031
6b Factor means nonverbal^a^	7	43.862	.939	.971	.091	[.067, .118]	–	–	–	–	–
6b versus 3	–	–	–	–	–	–	1	36.553	.059	.028	.145

*Note*. *N*_Total_ = 632, *N*_Without migration background_ = 316, *N*_With migration background_ = 316. CFI = Comparative Fit Index; Mc = McDonald’s Noncentrality Index; RMSEA = root mean square error of approximation; CI = confidence interval.

^a^Factor variance invariance and factor mean invariance were calculated at the level of scalar invariance.

Further, invariance of the latent variances and means for verbal and nonverbal intelligence (two-factor structure) were tested using the model implying scalar invariance as baseline (see Table 3). On the level of scalar invariance, results regarding verbal intelligence indicate that latent variances were invariant between groups with *s*^2^ = 117.20 for individuals with a migration background and *s*^2^ = 72.00 for individuals without a migration background. Latent means differed between groups and were lower for individuals with a migration background (*M* = 44.44) compared to individuals without a migration background (*M* = 53.05) with an effect size of *d* = 1.01, indicating a large effect. Regarding nonverbal intelligence, latent variances and latent means differed between groups. Latent variances were higher and latent means lower in individuals with a migration background (*s*^2^ = 107.40, *M* = 47.91) compared to individuals without a migration background (*s*^2^ = 72.81, *M* = 52.30). According to Cohen [47], the effect size of the group difference for nonverbal intelligence was medium with *d* = 0.52.

Results of the single-factor structure (Fig 1b) are presented in Table 4. Though configural and metric invariance held, the models implying scalar and residual invariance showed values of ΔCFI and ΔMc above the suggested cutoff values. Although the *p* value of the test of small difference in fit is < .05, we conclude that there are differences in subtests’ intercepts and residual variances across groups. As scalar invariance did not hold, we examined partial scalar invariance to identify specific subtests that caused noninvariance on the scalar level as proposed by Steenkamp and Baumgartner [41]. We found that the subtest OIO was the major contributor to the worsened model fit (S4 Table). Variances and means of general intelligence were not analyzed due to the lack of full scalar invariance [20].

**Table 4:** Fit Indices for Multi-group Confirmatory Factor Analysis Evaluating Measurement Invariance of the Single-factor Structure Including the Four Intelligence Subtests Across Individuals With and Without a Migration Background.

Model	*df*	χ^2^	CFI	Mc	RMSEA	90% CI	Δ*df*	Δχ^2^	ΔCFI	ΔMc	Small diff *p*
1 Configural invariance	3	11.506	.986	.993	.067	[.029, .110]	–	–	–	–	–
2 Metric invariance	6	16.481	.983	.992	.053	[.023, .084]	–	–	–	–	–
2 versus 1	–	–	–	–	–	–	3	4.975	.003	.001	.174
3 Scalar invariance	9	29.839	.965	.984	.061	[.037, .085]	–	–	–	–	–
3 versus 2	–	–	–	–	–	–	3	13.358	.018	.008	.271
4 Residual invariance	13	62.525	.918	.962	.078	[.059, .098]	–	–	–	–	–
4 versus 2	–	–	–	–	–	–	4	32.686	.047	.022	.181
5 Factor variances^a^	10	38.824	.952	.977	.068	[.046, .091]	–	–	–	–	–
5 versus 2	–	–	–	–	–	–	1	8.985	.013	.007	.270
6 Factor means^a^	10	150.616	.766	.895	.149	[.129, .171]	–	–	–	–	–
6 versus 2	–	–	–	–	–	–	1	120.777	.199	.089	.034

*Note*. *N*_Total_ = 632, *N*_Without migration background_ = 316, *N*_With migration background_ = 316. CFI = Comparative Fit Index; Mc = McDonald’s Noncentrality Index; RMSEA = root mean square error of approximation; CI = confidence interval.

^a^ Factor variance invariance and factor mean invariance were calculated at the level of measurement invariance, as scalar invariance did not hold.

## Discussion

The main goal of this study was to assess measurement invariance and differences in latent means of the German version of the RIAS in individuals with and without a migration background. We analyzed a two-factor structure with verbal and nonverbal intelligence as latent factors as well as a single-factor structure with general intelligence as the latent factor.

Both the two-factor and the single-factor structure were supported through single-CFAs for each group separately. This is in line with the RIAS technical manual [22, 24] and with previous research supporting the two-factor structure [30], the single-factor structure [23–26], or both RIAS intelligence factor structures [31, 32].

Then, we conducted multi-group CFAs to analyze measurement invariance for both structures across individuals with and without a migration background. For the two-factor structure, results showed scalar invariance across groups, indicating that differences in the subtest means were due to differences in the means of the underlying constructs [48]. Residual invariance could not be supported, possibly because of differing reliabilities of the scales across groups [49].

Further, we assessed invariance of latent variances and means of verbal and nonverbal intelligence. Results indicate that variances of nonverbal intelligence as well as means of verbal and nonverbal intelligence were noninvariant across groups. Individuals with a migration background showed similar variances in verbal intelligence and higher variances in nonverbal intelligence compared to individuals without a migration background. These results suggest that individuals with a migration background showed about the same range in verbal intelligence test scores but a wider range in nonverbal intelligence test scores than individuals without a migration background. Beaujean and McGlaughlin [34] found a similar pattern when examining referred black and white students, but black students showed a higher variance in general intelligence in the RIAS test scores. Further, as hypothesized, individuals with a migration background achieved lower latent means in verbal as well as nonverbal intelligence when tested with the German version of the RIAS. This is in line with other studies that also found lower means in intelligence test scores for individuals with a migration background [5, 7, 9]. For the group differences, the effect size in verbal intelligence was high, whereas the effect size in nonverbal intelligence was medium, according to Cohen [47]. This is in accordance with our hypothesis that differences in latent means would be especially evident in the RIAS VIX. A possible explanation for this result may be that language barriers in individuals with a migration background might have led to lower test performance especially in the RIAS subtests with high language requirements [6, 8, 9]. However, individuals with a migration background also showed lower test performance in nonverbal intelligence. Existing literature suggests that not only language barriers but also cultural and environmental differences such as familiarity with tasks used in intelligence tests [11, 12], stereotype threat [13, 18], and parental language skills [14] can contribute to lower test performance in individuals with a migration background [10], which also might have led to the lower test scores of individuals with a migration background in the verbal and nonverbal intelligence indexes in the present study. In sum, the results of the present study indicate that using the German version of the RIAS may lead to underestimated intelligence test scores in individuals with a migration background. Such an approach in which examiners focus exclusively on the test results is called a “static” intelligence assessment ([12], p. 445). In contrast, “dynamic” testing methods ([12], p. 445) may constitute an assessment approach that is able to account for differences in individuals from different cultural contexts. In line with the zone of proximal development concept [50], in dynamic testing, the tasks are first introduced to familiarize the individuals with the nature of these tasks. Afterward the improvement in these tasks is assessed to evaluate the learning potential of an examinee, which may be a better estimate of abilities compared to results gained from a static assessment [51]. Thus, there is evidence that dynamic testing leads to diminishing test score differences across individuals with and without a migration background [11, 12]. These results indicate that examiners in psychological practice should consider using a dynamic testing approach to reduce effects of familiarity with intelligence test tasks. It should be a goal for future studies to determine whether the positive effects of such a dynamic approach are also evident when using the German version of the RIAS in individuals with and without a migration background.

For the single-factor structure, only metric invariance and not scalar invariance held. This indicates that the relationship between the observed scores and the latent construct was different across groups [20]. Additionally, we assessed partial scalar invariance and found that the major contributor to differences across groups was the subtest OIO. This is in line with other studies that also found OIO to be the major contributor to a lack of full scalar invariance [30] or a decreased model fit of scalar invariance [34]. Thus, group differences in the latent means can be explained either through the mean differences in the subtest OIO or through true latent mean differences [48].

Because full scalar invariance did not hold for the single-factor structure, we did not analyze invariance of latent variances and means of general intelligence [21, 41]. This leads to the conclusion that test scores of individuals with a migration background should not be compared with test scores of individuals without a migration background on the level of general intelligence when tested with the RIAS, but rather on the level of verbal and nonverbal intelligence only.

There are strengths and limitations of this study. We see it as a strength that we analyzed competing factor structures (two-factor vs. single-factor) of the RIAS, and thus our results contribute to the discussion on the best-fitting RIAS intelligence factor structure for specific samples. Further, we analyzed large age- and sex-matched samples of individuals with and without a migration background. However, we could not control for social factors such as socioeconomic status (SES); previous studies have shown that SES is positively correlated with intelligence [52] and negatively related to migration background such that individuals with a migration background have a lower SES than individuals without a migration background [53, 54]. Therefore, it is possible that the means in individuals with a migration background would have been slightly higher if we additionally could have controlled for SES. Further, we determined migration background solely on the basis of individuals being foreign-language or native. Future studies could additionally gather the status of migration (country of birth, number of the family’s generations living in the country of immigration, etc.). Finally, our sample of individuals with a migration background included subjects with a diversity of first languages. Although this reflects the language diversity of Switzerland and Germany [2, 3], it can be assumed that these individuals do not represent a homogeneous group. Future studies might therefore focus on specific groups such as individuals from countries with collectivist or individualist cultural values [55] or from different language families [56]. Future studies might also analyze RIAS test scores in individuals with and without a migration background in other countries with a high percentage of migration.

## Conclusion

In conclusion, the RIAS two-factor intelligence structure including four subtests measuring verbal and nonverbal intelligence showed measurement invariance across samples, whereas we could only demonstrate partial scalar invariance for the single-factor structure with four subtests measuring general intelligence. These results suggest that examiners should focus on the VIX and NIX and not on the CIX when comparing RIAS test results of individuals with and without a migration background. Finally, examiners should consider the migration background as well as cultural, environmental, and social factors of an examinee when interpreting RIAS test results, as individuals with a migration background may achieve lower test scores in particular on the VIX but also on the NIX than individuals without a migration background, which may be due to language, cultural, environmental, or social barriers.

## Supporting Information

S1 Table Frequencies for Age Ranges for the Total Sample. (DOCX)

S2 Table Overview of All First Languages Named by Subjects With a Migration Background. (DOCX)

S3 Table Unstandardized and Standardized Factor Loadings, Covariances, and Correlations for the Two-Factor and Single-Factor Structures of the Confirmatory Factor Analyses for Individuals With and Without a Migration Background. (DOCX)

S4 Table Fit Indices for Multi-group Confirmatory Factor Analysis Evaluating Partial Measurement Invariance of the Single-factor Structure Including the Four Intelligence Subtests Across Individuals With and Without a Migration Background. (DOCX)
