# Supplementary material for: Measurement Invariance and Latent Mean Differences in the Reynolds Intellectual Assessment Scales (RIAS): Does the German Version of the RIAS Allow a Valid Assessment of Individuals with a Migration Background?
Source: PLoS One. 2016 Nov 15;11(11):e0166533. doi: 10.1371/journal.pone.0166533 (PMC5112777; doi:10.1371/journal.pone.0166533)
Supplement: S1 Table — (DOCX) [file pone.0166533.s001.docx]

**Supplemental Table 1. Frequencies for age ranges for the total sample.**

| Age range (years) | Frequency |
| --- | --- |
| 3-5 | 22.2% |
| 6-12 | 41.1% |
| 13-19 | 20.6% |
| 20-29 | 4.1% |
| 30-39 | 2.7% |
| 40-49 | 2.4% |
| 50-59 | 2.7% |
| 60-69 | 2.5% |
| 70-79 | 0.3% |
| 80-89 | 0.6% |
| 90-99 | 0.6% |

*Note. N* = 632.
