# Supplementary material for: Measurement Invariance and Latent Mean Differences in the Reynolds Intellectual Assessment Scales (RIAS): Does the German Version of the RIAS Allow a Valid Assessment of Individuals with a Migration Background?
Source: PLoS One. 2016 Nov 15;11(11):e0166533. doi: 10.1371/journal.pone.0166533 (PMC5112777; doi:10.1371/journal.pone.0166533)
Supplement: S2 Table — (DOCX) [file pone.0166533.s002.docx]

**Supplemental Table 2. Overview of all First Languages Named by Subjects With a Migration Background.**

| Language | Frequency |
| --- | --- |
| Albanian | 0.9% |
| Arabic | 1.9% |
| Bosnian | 2.2% |
| English | 4.7% |
| French | 0.6% |
| Hungarian | 0.3% |
| Greek | 0.3% |
| Indian | 3.2% |
| Italian | 7.9% |
| Croatian | 1.6% |
| Macedonian | 5.7% |
| Dutch | 0.3% |
| Persian | 0.3% |
| Polish | 1.3% |
| Portuguese | 4.7% |
| Romanian | 1.3% |
| Russian | 2.2% |
| Swedish | 1.3% |
| Serbian | 6.3% |
| Spanish | 5.4% |
| Tamil | 1.3% |
| Thai | 5.1% |
| Turkish | 20.6% |
| Ukrainian | 0.3% |
| Ukrainian and Turkish | 0.3% |

*Note. N*_With migration background_ = 316. A total of 19.9% of subjects did not answer this question.
