# Supplementary material for: Measurement Invariance and Latent Mean Differences in the Reynolds Intellectual Assessment Scales (RIAS): Does the German Version of the RIAS Allow a Valid Assessment of Individuals with a Migration Background?
Source: PLoS One. 2016 Nov 15;11(11):e0166533. doi: 10.1371/journal.pone.0166533 (PMC5112777; doi:10.1371/journal.pone.0166533)
Supplement: S3 Table — (DOCX) [file pone.0166533.s003.docx]

**Supplemental Table 3. Unstandardized and Standardized Factor Loadings, Covariances, and Correlations for the Two-Factor and Single-Factor Structures of the Confirmatory Factor Analyses for Individuals With and Without a Migration Background.**

| Structure | Unstandardized loadings (*SE*) | Standardized loadings | Covariance (*SE*) | Correlation |
| --- | --- | --- | --- | --- |
| Two factors^a^ |  |  |  |  |
| Without migration background |  |  |  |  |
| GWH (verbal) | 1.000 | .756 |  |  |
| VRZ (verbal) | 1.154 (.159) | .769 |  |  |
| OIO (nonverbal) | 1.000 | .641 |  |  |
| WHM (nonverbal) | 0.912 (.165) | .592 |  |  |
| Verbal–Nonverbal |  |  | 23.927 (4.309) | .659 |
| With migration background |  |  |  |  |
| GWH (verbal) | 1.000 | .761 |  |  |
| VRZ (verbal) | .889 (.102) | .852 |  |  |
| OIO (nonverbal) | 1.000 | .850 |  |  |
| WHM (nonverbal) | .755 (.104) | .666 |  |  |
| Verbal–Nonverbal |  |  | 45.384 (7.166) | .585 |
| Single factor^b^ |  |  |  |  |
| Without migration background |  |  |  |  |
| GWH (g) | 1.000 | .595 |  |  |
| VRZ (g) | 1.172 (.186) | .615 |  |  |
| OIO (g) | .832 (.163) | .469 |  |  |
| WHM (g) | .737 (.153) | .420 |  |  |
| e1 – e2 |  |  | 19.092 (2.438) | .365 |
| e3 – e4 |  |  | 19.092 (2.438) | .297 |
| With migration background |  |  |  |  |
| GWH (g) | 1.000 | .633 |  |  |
| VRZ (g) | .870 (.092) | .692 |  |  |
| OIO (g) | .982 (.124) | .658 |  |  |
| WHM (g) | .773 (.109) | .539 |  |  |
| e1 – e2 |  |  | 19.092 (2.438) | .345 |
| e3 – e4 |  |  | 19.092 (2.438) | .282 |

*Note. N*_Without migration background_ = 316, *N*_With migration background_ = 316. *SE* = Standard error; GWH = Guess What; VRZ = Verbal Reasoning; OIO = Odd-Item Out; WHM = What’s Missing; g = general intelligence; e1 – e2 = residuals verbal; e3 – e4 = residuals nonverbal.

^a^Includes four subtests with loadings on the verbal factor (GWH, VRZ) and nonverbal factor (OIO, WHM).

^b^Includes four subtests with loadings on the g factor (GWH, VRZ, OIO, WHM)
