# Supplementary material for: Measurement Invariance and Latent Mean Differences in the Reynolds Intellectual Assessment Scales (RIAS): Does the German Version of the RIAS Allow a Valid Assessment of Individuals with a Migration Background?
Source: PLoS One. 2016 Nov 15;11(11):e0166533. doi: 10.1371/journal.pone.0166533 (PMC5112777; doi:10.1371/journal.pone.0166533)
Supplement: S4 Table — (DOCX) [file pone.0166533.s004.docx]

**Supplemental Table 4. Fit Indices for Multi-group Confirmatory Factor Analysis Evaluating Partial Measurement Invariance of the Single-factor Structure Including the Four Intelligence Subtests Across Individuals With and Without a Migration Background.**

| Model | *df* | χ^2^ | CFI | Mc | RMSEA | 90% CI | Δ*df* | Δχ^2^ | ΔCFI | ΔMc | Small diff *p* |
| --- | --- | --- | --- | --- | --- | --- | --- | --- | --- | --- | --- |
| 1 Configural invariance | 3 | 11.506 | .986 | .993 | .067 | [.029, .110] | – | – | – | – | – |
| 2 Metric invariance | 6 | 16.481 | .983 | .992 | .053 | [.023, .084] | – | – | – | – | – |
| 2 versus 1 | – | – | – | – | – | – | 3 | 4.975 | .003 | .001 | .354 |
| 3 Scalar invariance | 8 | 21.876 | .977 | .989 | .052 | [.027, .079] | – | – | – | – | – |
| 3 versus 2 | – | – | – | – | – | – | 3 | 5.395 | .005 | .003 | .282 |
| 4 Residual invariance | 12 | 54.404 | .929 | .967 | .075 | [.055, .096] | – | – | – | – | – |
| 4 versus 3 | – | – | – | – | – | – | 4 | 32.528 | .048 | .022 | .189 |
| 5 Factor variances^a^ | 9 | 30.737 | .964 | .977 | .062 | [.039, .087] | – | – | – | – | – |
| 5 versus 3 | – | – | – | – | – | – | 1 | 8.861 | .013 | .007 | .284 |
| 6 Factor means^a^ | 9 | 150.612 | .765 | .895 | .158 | [.136, .181] | – | – | – | – | – |
| 6 versus 3 | – | – | – | – | – | – | 1 | 128.736 | .212 | .089 | .034 |

*Note. N*_Total_ = 632, *N*_Without migration background_ = 316, *N*_Migration background_ = 316. CFI = Comparative Fit Index; Mc = McDonald’s Noncentrality Index; RMSEA = root mean square error of approximation; CI = confidence interval. Intercept of OIO was freely estimated.

^a^ Factor variance invariance and factor mean invariance were calculated at the level of scalar invariance.
